# Validation of Optimal Reference Genes for qRT-PCR in Adipose- and Uterine-Derived Feline Mesenchymal Stem Cells

**DOI:** 10.3390/vetsci13090902

**Published:** 2026-09-02

**Authors:** Rubel Miah, Sang-Yun Lee, Su Hyeon Song, Dong-Ju Park, Yong-Ho Choe, Sung-Lim Lee, Won-Jae Lee, Chan-Hee Jo, Eun-Yeong Bok, Hyeon-Jeong Lee, Yong-Xun Jin, Yeon-Woo Jeong, Young-Bum Son

**Affiliations:** 1 Department of Obstetrics, College of Veterinary Medicine, Chonnam National University, 300 Yonbongdong, Buk-gu, Gwangju 61186, Republic of Korea; rubelsau26@gmail.com (R.M.); sy0lee@knue.ac.kr (S.-Y.L.); akasla123@jnu.ac.kr (S.H.S.); 2 Department of Biology Education, Korea National University of Education, Cheongju 28173, Republic of Korea; 3 Institute of Agricultural and Life Science, Division of Animal Bioscience and Integrated Biotechnology, Gyeongsang National University, Jinju 52828, Republic of Korea; djpark@gnu.ac.kr; 4 Department of Theriogenology and Biotechnology, College of Veterinary Medicine, Gyeongsang National University, Jinju 52628, Republic of Korea; yhchoe@gnu.ac.kr (Y.-H.C.); sllee@gnu.ac.kr (S.-L.L.); ch_jo@gnu.ac.kr (C.-H.J.); 5 Department of Obstetrics, College of Veterinary Medicine, Kyungpook National University, Daegu 41566, Republic of Korea; iamcyshd@knu.ac.kr; 6 Division of Animal Diseases & Health, National Institute of Animal Science, Rural Development Administration, Wanju 55365, Republic of Korea; eybok@korea.kr; 7 Korea Advanced Medical Training Institute, Daegu-Gyeongbuk Medical Innovation Foundation, Daegu 41061, Republic of Korea; leehj@kmedihub.re.kr; 8 Jilin Provincial Key Laboratory of Animal Model, College of Animal Science, Jilin University, Changchun 130062, China; jyx0429@126.com; 9 Department of Companion Animal and Animal Resources Science, Joongbu University, Geumsan 32713, Republic of Korea; 10 Animal Medical Institute, Chonnam National University, Gwangju 61186, Republic of Korea

**Keywords:** feline, reference gene, adipose tissue, uterine tissue, mesenchymal stem cells

## Abstract

Feline mesenchymal stem cells (MSCs) isolated from adipose (A-MSCs) and uterine (U-MSCs) tissues during routine ovariohysterectomy represent valuable therapeutic sources in veterinary regenerative medicine. While quantitative real-time PCR (qRT-PCR) remains the gold standard for transcriptional profiling in these cells, its analytical reliability relies on empirically validated reference genes. In this study, we assessed the expression stability of nine candidate reference genes (*ACTB*, *B2M*, *GAPDH*, *GUSB*, *HMBS*, *HPRT1*, *RPL7*, *TBP*, and *YWHAZ*) in feline A-MSCs and U-MSCs using the geNorm and NormFinder algorithms. Both computational approaches identified *TBP* and *HMBS* as the most stably expressed genes across both cell types, whereas the historically popular *GAPDH* was the least stable. All geNorm pairwise variation values were below the commonly used threshold of 0.15 in both A-MSCs and U-MSCs. To illustrate the impact of reference gene choice, we evaluated the pluripotency marker *OCT4*. Normalizing using *TBP* and *HMBS* revealed significant differences in *OCT4* expression between A-MSCs and U-MSCs (*p* < 0.05). In contrast, applying the unstable *GAPDH* masked this difference. These findings identify *TBP* and *HMBS* as promising reference gene candidates for qRT-PCR normalization in P3 undifferentiated feline A-MSCs and U-MSCs.

## Simple Summary

Gene expression analysis is essential for evaluating feline mesenchymal stem cells (MSCs). While quantitative real-time PCR (qRT-PCR) is a standard tool for this purpose, its accuracy depends on selecting stable reference genes for data normalization. The routine use of traditional reference genes without tissue-specific validation introduces severe analytical bias. Therefore, we evaluated nine candidate reference genes in feline MSCs isolated from adipose and uterine tissues. *HMBS* and *TBP* were the most stably expressed genes across both cell populations. Conversely, *GAPDH* exhibited the highest variability. To illustrate the impact of reference gene selection, we demonstrated that normalizing target gene expression against an unvalidated control can mask true biological differences between these cells. In conclusion, *HMBS* and *TBP* were identified as the most stably expressed reference gene candidates among the nine genes for evaluating gene expression using qRT-PCR in P3 undifferentiated feline A-MSCs and U-MSCs under the normal culture conditions in this study.

## 1. Introduction

Mesenchymal stem cells (MSCs) are widely used in veterinary regenerative medicine due to their inherent self-renewal capacity and multilineage differentiation potential [1,2]. In feline regenerative medicine, MSC therapies are being actively investigated for the treatment of refractory immune-mediated and inflammatory conditions, including feline chronic gingivostomatitis (FCGS) and chronic kidney disease (CKD) [3,4]. Despite these clinical advancements, securing a reliable and consistent cell source remains a challenge [5]. Routine ovariohysterectomy (OHE) provides an ethical and accessible solution [6,7], allowing for the collection of multiple tissue types, including adipose and uterine tissues, without requiring additional invasive procedures [7]. Both adipose tissue- and uterine tissue- derived MSCs obtained during OHE represent valuable cell sources for regenerative research [8,9].

Due to its analytical sensitivity and specificity, quantitative real-time PCR (qRT-PCR) remains the gold standard for evaluating gene expression profiles in isolated MSCs [10,11]. However, achieving quantitative accuracy requires normalization against stably expressed reference genes [12]. The use of unvalidated reference genes can introduce bias into the data, potentially leading to erroneous interpretations [13]. To address this, the Minimum Information for Publication of Quantitative Real-Time PCR Experiments (MIQE) guidelines require the empirical validation of reference genes prior to data normalization [14]. Despite these established standards and the increasing clinical use of feline MSCs, the systematic validation of reference genes for feline tissues remains scarce, particularly for MSCs derived from OHE [15]. Many regenerative studies use unvalidated conventional housekeeping genes, such as *GAPDH* or *ACTB* [16]. Biologically, however, adipose and uterine tissues have distinct physiological functions. The adipose tissue-derived MSCs exhibit specific metabolic activity related to lipid storage, whereas uterine tissue-derived MSCs are continuously subjected to dynamic immunological and hormonal remodelling. Consequently, this practice can introduce systematic bias, as the transcription levels of these traditional genes can fluctuate in response to tissue-specific metabolic demands, hormonal shifts, and distinct physiological or immune states [17,18].

Therefore, the present study aims to identify stably expressed reference genes for qRT-PCR normalization in feline adipose tissue- and uterine tissue-derived MSCs. To comply with assessment standards, we evaluated the expression stability of nine candidate genes (*GAPDH*, *ACTB*, *GUSB*, *RPL7*, *HPRT1*, *TBP*, *YWHAZ*, *B2M*, and *HMBS*) using two established statistical algorithms, geNorm [19] and NormFinder [20]. geNorm determines stability by calculating pairwise variation, whereas NormFinder utilizes an analysis of variance (ANOVA)-based mathematical model to assess intra- and inter-group variations. Employing these distinct statistical approaches simultaneously prevents single-algorithm bias and provides a comprehensive consensus ranking [20]. Furthermore, to demonstrate the impact of reference gene selection, we analyzed how the expression pattern of the pluripotency marker *OCT4* [21] shifts when normalized against the most stable versus the least stable candidate genes.

## 2. Materials and Methods

### 2.1. Ethics Approval and Animals

All procedures were approved by the research ethics committee of Chonnam National University Animal Centre for Biomedical Experimentation (CNU IACUC-YB-2025-73). Adipose and uterine tissues were collected from four female cats during ovariohysterectomy.

### 2.2. Chemicals and Media

Unless explicitly stated otherwise, all chemicals were purchased from Sigma (St. Louis, MO, USA), and all media were obtained from Gibco (Gibco Life Technologies, Gaithersburg, MD, USA).

### 2.3. Establishment and Culture of MSCs from Adipose and Uterine Tissues

Adipose and uterine tissues were collected from four female cats undergoing routine OHE. MSCs were isolated and expanded from both tissue types following a previously described protocol, with minor modifications [22]. Briefly, the tissues were finely minced and enzymatically digested with 0.1% collagenase type I at 37 °C with gentle agitation. The digested homogenate was then filtered through a 100 μm cell strainer to remove undigested structural debris. The suspended cells were centrifuged at 300× *g* for 5 min. The cell pellet was subsequently resuspended in Advanced Dulbecco’s Modified Eagle’s Medium (ADMEM) supplemented with 20% fetal bovine serum (FBS), 1% penicillin–streptomycin, and 1% GlutaMAX. The isolated cells were seeded into culture flasks and maintained in a humidified atmosphere at 37 °C with 5% CO_2_. Upon reaching 80–90% confluence, the adherent cells were detached using 0.25% trypsin-EDTA, harvested via centrifugation (300× *g*, 5 min), and subcultured. To minimize potential passage-associated changes such as changes in gene expression and cellular characteristics while allowing sufficient cell expansion, cells at passage 3 were exclusively used for all subsequent experiments.

### 2.4. Cell Surface Marker Analysis Using Flow Cytometry

To analyze the expression of MSC surface markers, cells were harvested using 0.25% trypsin–EDTA and fixed with 4% paraformaldehyde for an hour. Following fixation, the cells were incubated with FITC-conjugated mouse IgG1 κ isotype control (BD Biosciences, Frankline Lakes, NJ, USA), APC-conjugated mouse IgG1 κ isotype control (BD Biosciences), FITC-conjugated mouse anti-human CD34 (BD Biosciences), FITC-conjugated rat anti-mouse CD44 (BD Biosciences), FITC-conjugated rat anti-dog CD45 (Thermo Fisher Scientific, Rockford, IL, USA), FITC-conjugated mouse anti-human CD90 (BD Biosciences), and APC-conjugated mouse anti-human CD105 (Thermo Fisher Scientific) at 4 °C for 1 h in the dark. Data acquisition was conducted using a FACSLyric™ flow cytometer (BD Biosciences). Gating strategies were established based on the fluorescence characteristics of the corresponding isotype controls to exclude non-specific binding. Finally, data analysis and quantification of surface marker expression were performed using FlowJo™ software v10.

### 2.5. In Vitro Multilineage Differentiation of MSCs

To assess the multilineage differentiation capacity of MSCs, cells were subjected to adipogenic and osteogenic induction. For adipogenic differentiation, MSCs were cultured for 21 days in DMEM supplemented with 10% fetal bovine serum (FBS), 1% penicillin–streptomycin (P/S), 1 µM dexamethasone, 100 µM indomethacin, and 10 µM insulin. Intracellular lipid droplet accumulation was visualized using Oil Red O staining. For osteogenic differentiation, cells were maintained for 21 days in DMEM enriched with 10% FBS, 1% P/S, 200 µM ascorbic acid, 0.1 µM dexamethasone, and 10 mM β-glycerophosphate. Matrix mineralization and calcium deposition were subsequently confirmed via Alizarin Red S staining. All differentiation outcomes were evaluated and imaged using a bright-field microscope.

### 2.6. Selection of Reference Genes and Primers

We selected nine reference genes based on previous reports and their diverse intracellular functions [23,24]. Specific primer pairs for each gene were designed using Primer3 software. To ensure optimal amplification, the absence of secondary structures, including homodimers, heterodimers, and hairpins, was verified using OligoAnalyzer 3.1 software. The gene names, nucleotide sequences, and expected amplicon sizes are detailed in Table 1. To assess the amplification efficiency of each primer pair, standard curves were constructed using Ct values derived from a four-fold serial dilution of representative cDNA samples. The correlation coefficient (R^2^), slope (M), intercept (B), and PCR efficiency (E) for each target were calculated using qPCRsoft 4.1 software (Analytik Jena, Jena, Germany).

### 2.7. RNA Extraction, cDNA Synthesis, and qRT-PCR

Total RNA was extracted from MSCs using the easy-spin™ Total RNA Extraction Kit (iNtRON Biotechnology, Seongnam, Republic of Korea). RNA concentration and purity based on the 260/280 ratio were assessed using an OPTIZEN™ NanoQ spectrophotometer (KLAB, Daejeon, Republic of Korea). For cDNA synthesis, 250 ng of total RNA was reverse transcribed using the ReverTra Ace™ qPCR RT Master Mix (TOYOBO, Osaka, Japan) at 37 °C for 1 h, following the manufacturer’s instructions. For qRT-PCR, the reaction mixture was prepared by combining 50 ng of cDNA, 0.1 μM of each forward and reverse primer, and THUNDERBIRD™ SYBR^®^ qPCR Mix (TOYOBO). Amplification was performed using a qTOWER^3^ real-time PCR thermal cycler (Analytik Jena, Jena, Germany). The thermal cycling conditions consisted of an initial activation step at 95 °C for 12 min, followed by 40 cycles of denaturation at 95 °C for 15 s, annealing at 60 °C for 15 s, and extension at 72 °C for 30 s. No-template controls were included in each qRT-PCR run to monitor contamination and non-specific amplification. Cycle threshold (Ct) values and melting curves were acquired and processed using qPCRsoft 4.1 software (Analytik Jena). The amplified PCR products were examined by gel electrophoresis to confirm the expected amplicon sizes.

### 2.8. Stable Reference Gene Expression Analysis

The stability of the nine candidate reference genes was evaluated using geNorm and NormFinder. The geNorm algorithm calculates an expression stability value (M) for each gene. Genes exhibiting the highest M values were sequentially excluded until only the two most stable candidates remained. Additionally, geNorm computes the pairwise variation (Vn/n+1) between sequential normalization factors (NFs) to assess whether the inclusion of an additional reference gene is warranted [19]. To avoid incorporating an unnecessary number of reference genes, a Pearson correlation analysis was conducted using GraphPad Prism version 10 software to compare NF_2_ (derived from the two most stable genes) with NF_opt_ (based on the theoretically optimal gene count). Furthermore, using an ANOVA model, NormFinder calculates stability values that reflect both intra- and inter-group variation. This algorithm ranks the candidate genes, identifying the single most stable reference gene indicated by the lowest stability score while also suggesting the optimal gene pair for overall normalization [20].

### 2.9. Normalization of GOI Using Different Reference Genes

To investigate the effect of reference gene stability on target gene quantification, the relative expression of the pluripotency marker *OCT4* was analyzed. Normalization of the *OCT4* transcripts was performed independently using the most stable reference gene and the least stable candidate gene identified in the current study. The relative mRNA expression levels were calculated using the 2^−ΔΔCt^ method. The qRT-PCR reactions were conducted as described previously, and data quantification was processed using qPCRsoft 4.1 software.

### 2.10. Statistical Analysis

All statistical evaluations were conducted using GraphPad Prism version 8.0. Because A-MSCs and U-MSCs were derived from the same individual cats, comparisons between the two cell types were performed using a paired Student’s *t*-test. The results are expressed as the mean ± standard deviation (SD) of the four biological replicates, with A-MSCs and U-MSCs established from four individual cats, each analyzed in three technical replicates. The technical replicates were averaged before statistical analysis. Statistical significance was defined as *p*-values of less than 0.05.

## 3. Results

### 3.1. Characterization of Feline A-MSCs and U-MSCs

**Figure 1:**
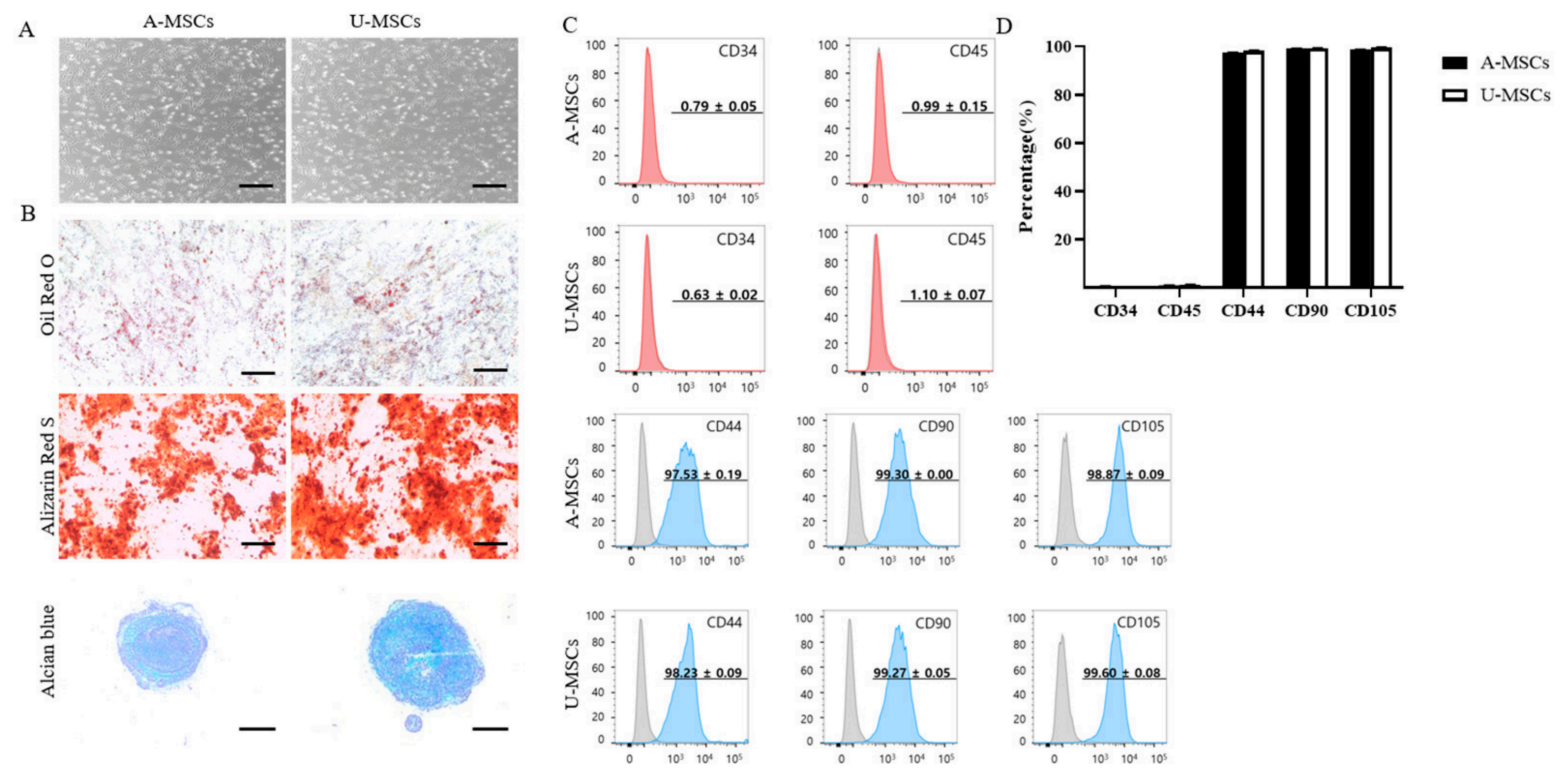
Characterization of feline adipose- and uterine-derived mesenchymal stem cells (A-MSCs and U-MSCs). (**A**) Representative phase-contrast images showing the spindle-like morphology of cultured A-MSCs and U-MSCs (scale bar = 200 µm). (**B**) Differentiation potential was evaluated by lineage-specific cytochemical staining. Oil Red O staining demonstrates intracellular lipid droplet accumulation in adipocytes. Alizarin Red S staining demonstrates calcium deposition in osteocytes. (**C**) Immunophenotypic analysis by flow cytometry revealed positive expression of CD44, CD90, and CD105 and negative expression of CD34 and CD45 in both A-MSCs and U-MSCs. Grey peaks show isotype IgG expression as a control. The CD markers are represented by red or blue peaks. (**D**) Flow cytometry revealed that MSC-specific surface markers were positively expressed and hematopoietic markers were negatively expressed in A-MSCs and U-MSCs.

MSCs were isolated from feline adipose and uterine tissues. The adipose tissue-derived MSCs (A-MSCs) and uterine tissue-derived MSCs (U-MSCs) cultured in standard growth media exhibited a spindle-like morphology (Figure 1A). Under specific differentiation conditions, both A-MSCs and U-MSCs differentiated into adipocytes and osteocytes. Adipogenic differentiation was verified by the presence of intracellular lipid droplets stained with Oil Red O (Figure 1B). Osteogenic differentiation was confirmed by mineralized nodules stained with Alizarin Red S (Figure 1B). Flow cytometry analysis revealed that both A-MSCs and U-MSCs were positive for the MSC-specific surface markers CD44, CD90, and CD105, but negative for the hematopoietic markers CD34 and CD45 (Figure 1C,D).

### 3.2. Evaluation of Amplicon Size, Primer Efficiency, and Ct Values of Candidate Reference Genes

**Figure 2:**
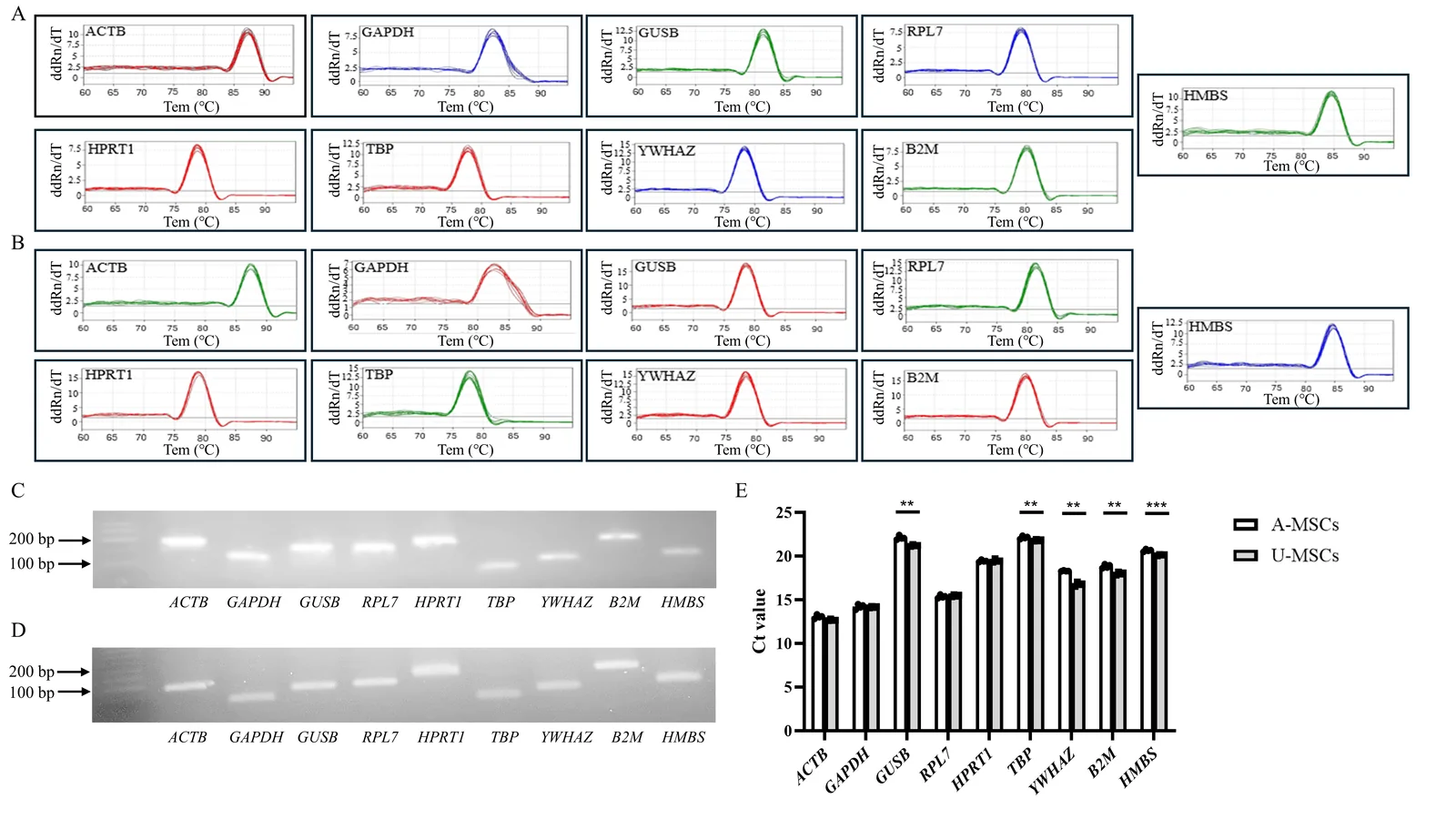
Assessment of primer specificity, amplicon size, and Ct values of the candidate reference genes in A-MSCs and U-MSCs. (**A**,**B**) Melting curve analysis of the nine candidate reference genes in (**A**) A-MSCs and (**B**) U-MSCs. A single peak was detected for each primer set, indicating specific amplification without nonspecific products or primer-dimer formation. (**C**,**D**) RT-qPCR amplicons were examined by 2% agarose gel electrophoresis in (**C**) A-MSCs and (**D**) U-MSCs. All primer pairs displayed a single band at the expected amplicon size. (**E**) Distribution of Ct values for the nine candidate reference genes in A-MSCs and U-MSCs. Data are presented as the mean ± SD. Statistical comparisons were performed between A-MSCs and U-MSCs for each candidate reference gene (** *p* < 0.01, *** *p* < 0.001).

To determine amplification efficiency, four-fold serial dilutions of cDNA were used to generate standard curves. The amplification efficiency (E) values for all candidate primer sets ranged from 0.99 to 1.02, with correlation coefficients (R^2^) exceeding 0.984, indicating reliable qPCR amplification (Table 1). Melt curve analysis confirmed primer specificity, as each primer pair generated a single distinct peak without primer-dimers or non-specific products (Figure 2A,B). Gel electrophoresis further revealed single bands matching the predicted amplicon sizes for all genes (Figure 2C,D). Cycle threshold (Ct) values were analyzed to assess the relative transcript abundance and basal expression variation of the candidate reference genes across all samples (Figure 2E).

### 3.3. Profiling of Expression Stability Using geNorm Algorithmic Ranking

**Figure 3:**
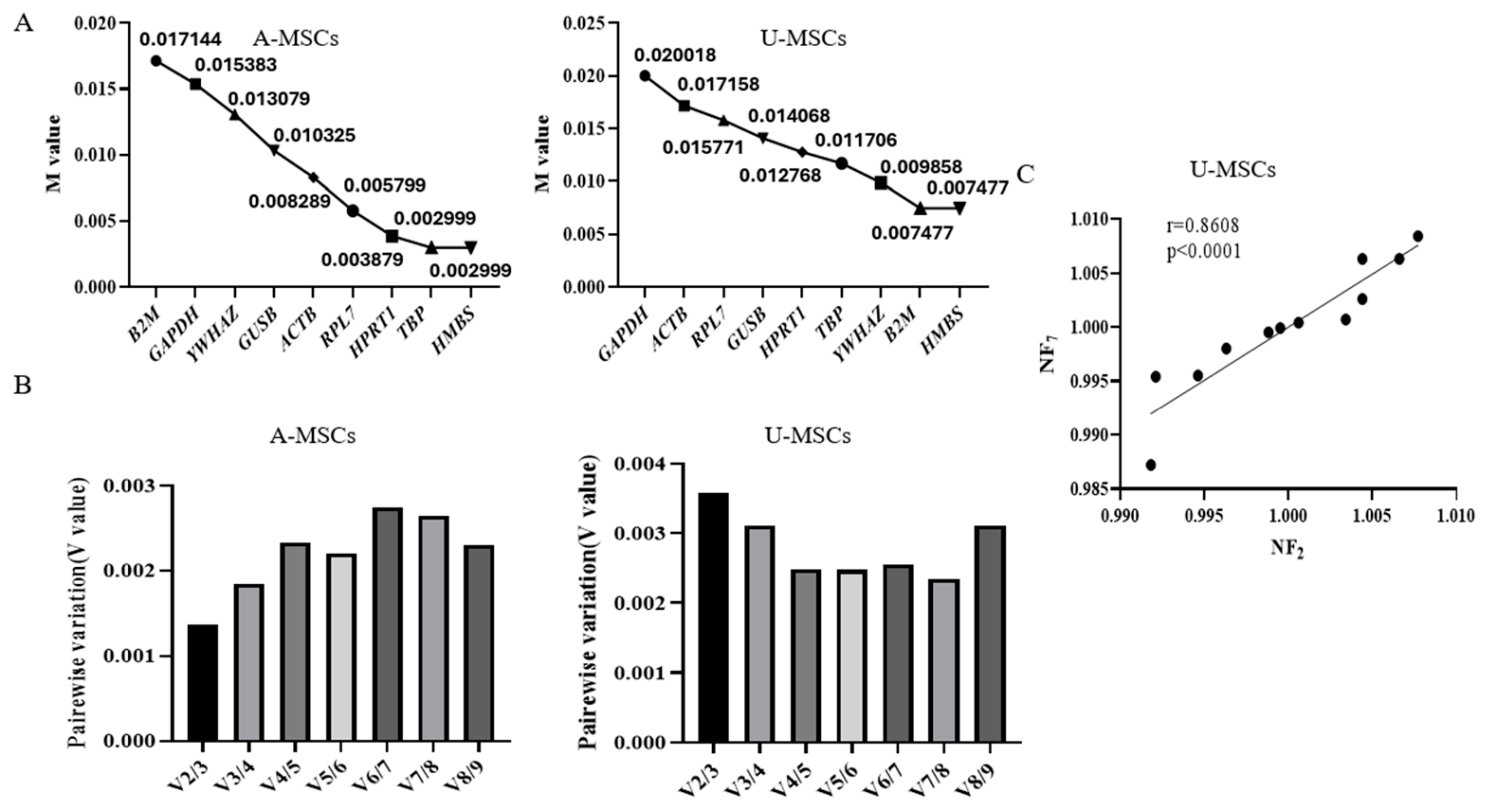
Assessment of expression stability, pairwise variation, and normalization factors of candidate reference genes using geNorm in A-MSCs and U-MSCs. (**A**) Average expression stability M values and ranking of the candidate reference genes in A-MSCs and U-MSCs. Lower M values indicate greater expression stability. The different symbols used on the plot lines are solely for distinguishing each candidate reference gene and do not carry additional statistical or biological meaning (**B**) Pairwise variation (V) analysis of sequential normalization factors in A-MSCs and U-MSCs. The minimum V values were observed at V_2/3_ in A-MSCs and V_7/8_ in U-MSCs. (**C**) Pearson’s correlation analysis between NF_7_ and NF_2_ in U-MSCs. A strong positive correlation was observed in U-MSCs (*r* = 0.8608, *p* < 0.001).

The average expression stability values (M) for nine candidate reference genes were calculated using the geNorm algorithm. All candidate genes showed M values below the default threshold of 1.5. In A-MSCs, *RPL7*, *HPRT1*, *TBP*, and *HMBS* were the most stable genes, whereas *GAPDH* was the least stable. For U-MSCs, *TBP*, *YWHAZ*, *B2M*, and *HMBS* exhibited the highest stability. Across both cell types, *GAPDH* consistently showed the lowest stability (Figure 3A). Subsequently, pairwise variation (V) was calculated to determine the optimal number of reference genes required for reliable normalization. Pairwise variation analysis revealed that all V values were below 0.004 in both A-MSCs and U-MSCs, substantially lower than the commonly used geNorm threshold of 0.15. The minimum V values were achieved at V_2/3_ (V = 0.001379) for A-MSCs, and at V_7/8_ (V = 0.002343) for U-MSCs (Figure 3B). Subsequently, we assessed the correlation between the normalization factors of the top two genes (NF_2_) and the top seven genes (NF_7_) in U-MSCs. A strong positive correlation was observed in U-MSCs (r = 0.8608, *p* < 0.001) (Figure 3C).

### 3.4. Analysis of Reference Gene Stability Using NormFinder

**Figure 4:**
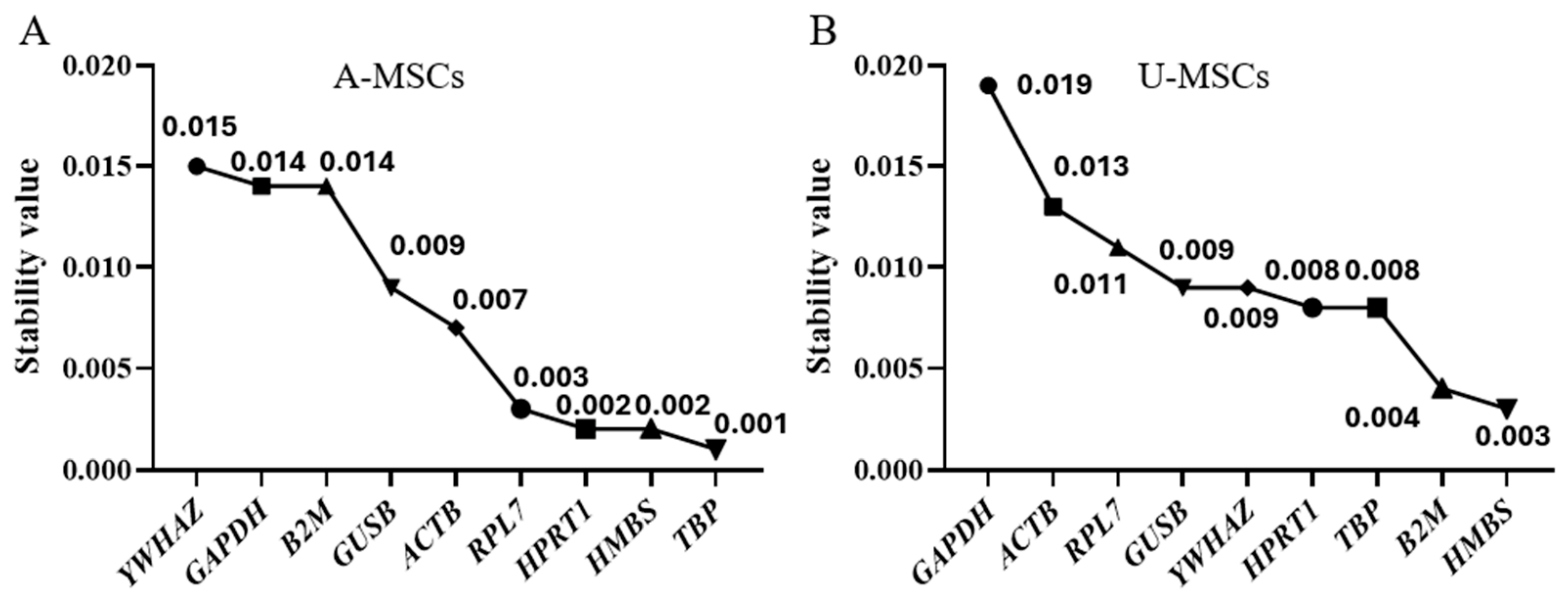
Assessment of reference gene stability using NormFinder in A-MSCs and U-MSCs. (**A**) The most stable reference gene is shown on the right side and the most unstable reference gene on the left side of the graph in A-MSCs. (**B**) The stability ranking of potential reference genes in U-MSCs is displayed in the graph. Lower stability values indicate greater expression stability. The different symbols used on the plot lines are solely for distinguishing each candidate reference gene and do not carry additional statistical or biological meaning.

NormFinder analysis was performed to evaluate the expression stability of the nine candidate genes. In A-MSCs, *RPL7*, *HPRT1*, *HMBS*, and *TBP* were identified as the most stable genes. In U-MSCs, *HPRT1*, *TBP*, *HMBS*, and *B2M* showed the highest stability. These results showed *TBP* and *HMBS* as the most stable candidate reference genes across both A-MSCs and U-MSCs. In contrast, *GAPDH* showed the lowest stability in both A-MSCs and U-MSCs, indicating that it is unsuitable for reliable normalization (Figure 4).

### 3.5. Impact of Reference Gene Stability on OCT4 Normalization

**Figure 5:**
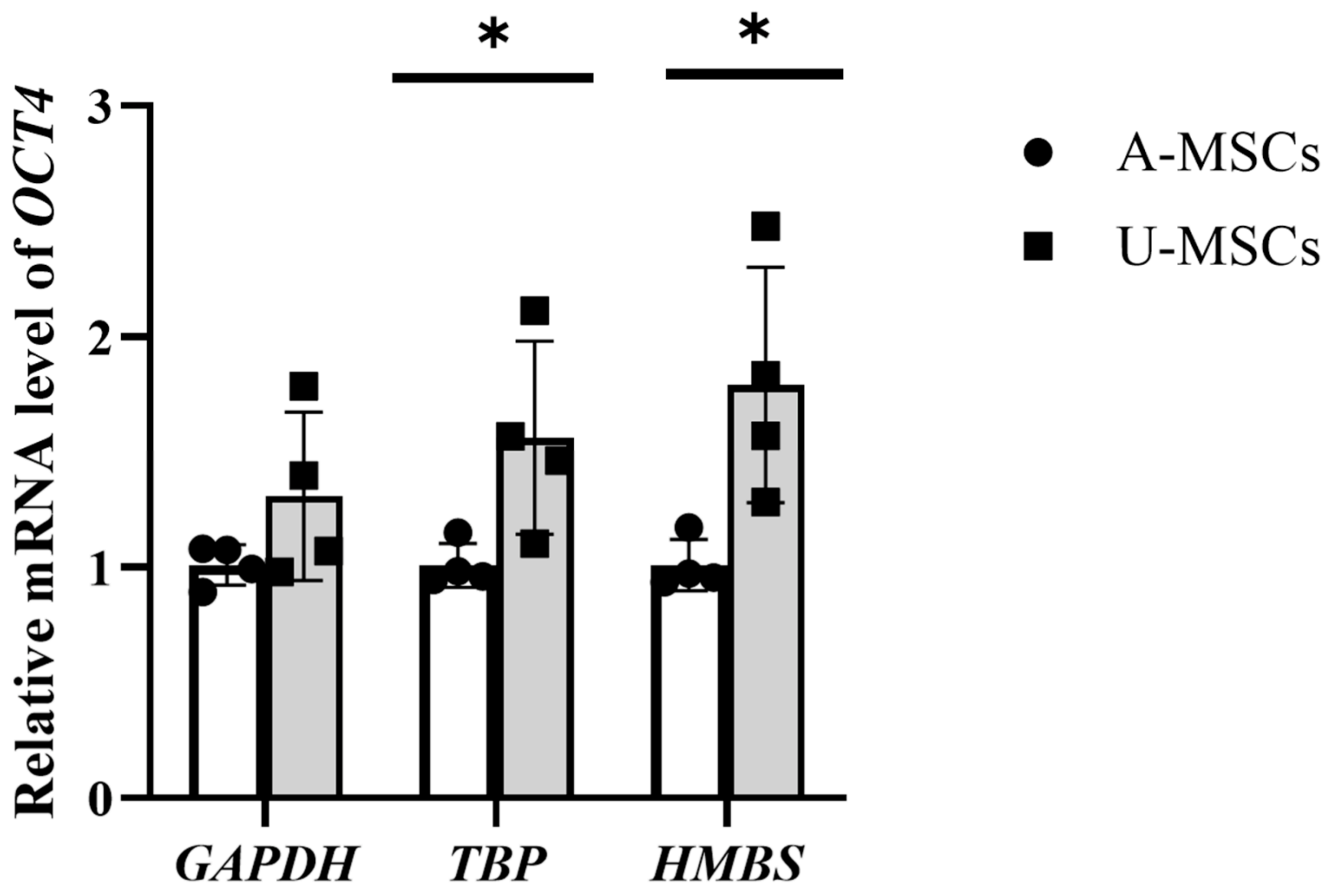
Effect of reference gene selection on the normalization of *OCT4* expression in A-MSCs and U-MSCs. *OCT4* mRNA expression was normalized using the two stable reference genes (*TBP* and *HMBS*) or the least stable reference gene (*GAPDH*). Data are presented as the mean ± SD. Differences between A-MSCs and U-MSCs that were statistically significant are displayed with asterisks (* *p* < 0.05).

To evaluate the effect of reference gene selection on target gene quantification, the expression of the pluripotency marker *OCT4* was analyzed. Normalization was performed using either the most stable gene panel including *TBP* and *HMBS* or the least stable gene such as *GAPDH*. While normalization with *TBP* and *HMBS* revealed a significant difference in *OCT4* expression between A-MSCs and U-MSCs (*p* < 0.05), this statistical difference was masked when *GAPDH* was used (Figure 5).

## 4. Discussion

MSCs derived from feline adipose and uterine tissues are considered promising resources for regenerative veterinary medicine [6,25]. However, the accurate quantification of target gene expression in these cells depends on normalization against stably expressed reference genes. While reference gene stability has been widely reported in human and livestock models [26,27,28], comprehensive studies identifying optimal internal controls in feline MSCs remain scarce. To address this, the present study used A-MSCs and U-MSCs harvested from female cats following routine OHE, providing a reliable platform for identifying stable internal controls across different tissue origins.

Prior to evaluating reference gene stability, the isolated A-MSCs and U-MSCs were analyzed to confirm their biological characteristics (Figure 1). Under defined culture conditions, both feline MSC populations exhibited spindle-like morphology, expression of MSC-specific surface markers, and the potential for multilineage differentiation. These results confirmed that the isolated cells possessed the fundamental characteristics of MSCs, ensuring the reliability of subsequent gene expression analyses.

The cell passage number is an important factor in MSC-based gene analysis studies because prolonged cell culture could alter cellular and transcriptional characteristics. In a previous study, it was demonstrated that early passage porcine umbilical cord MSCs retained stronger stemness-related characteristics than late passage cells [29]. In the present study, all experiments were conducted using P3 cells to minimize potential passage-associated changes.

Reliable qPCR analysis requires high-quality RNA, appropriate primer design, and suitable amplicon size to ensure amplification consistency [30]. In the present study, RNA purity was assessed spectrophotometrically. All primer pairs were designed to generate amplicons ranging from 80 to 190 base pairs, and qPCR assay performance was supported by acceptable amplification efficiencies, single melting-curve peaks, and single bands of the expected size on agarose gels. However, RNA integrity was not independently quantified using an electrophoretic integrity metric. Therefore, the possible effects of RNA integrity variation on Ct values and reference gene stability cannot be completely excluded, and this limitation should be addressed in future validation studies in accordance with MIQE recommendations.

To determine the most suitable internal controls, we used two widely adopted algorithms, geNorm and NormFinder [31]. Although geNorm and NormFinder apply different mathematical frameworks to the same expression dataset and may yield minor differences in stability rankings [32], the two algorithms showed concordant results, with *HMBS* and *TBP* as the most stably expressed genes. Based on the pairwise variation analysis, all V values were below 0.004 in both A-MSCs and U-MSCs, which is substantially lower than the commonly used geNorm threshold of 0.15. The addition of a third reference gene was not indicated according to the conventional geNorm criterion. In U-MSCs, the lowest V value, V_7/8_, was interpreted as a further reduction in pairwise variation rather than as a requirement for seven reference genes. We evaluated the correlation between the normalization factors of the two (NF_2_) and seven (NF_7_) genes. A strong positive correlation (r = 0.8608) was observed between NF_2_ and NF_7_. However, this correlation was considered complementary descriptive evidence and was not interpreted as evidence of equivalence or interchangeability between the two normalization strategies. Previous studies have suggested that the use of two or three validated reference genes can provide reliable normalization results [33,34]. Therefore, pairwise variation analysis using geNorm is important for evaluating whether the inclusion of additional reference genes is warranted for normalization under a given experimental condition.

Among the nine evaluated candidate reference genes in A-MSCs and U-MSCs, *HMBS* and *TBP* exhibited the highest expression stability. Conversely, *GAPDH* was determined to be the least stable internal control. TATA-box binding protein (*TBP*) functions as a critical component for eukaryotic transcription initiation, directing core promoter recognition and the assembly of the pre-initiation complex [35]. Consistent with our results, *TBP* has also been validated as a reliable reference gene in feline MSCs derived from the ovary and testis [36]. Similarly, hydroxymethylbilane synthase (*HMBS*) is a fundamental enzyme in the heme biosynthesis pathway, catalyzing the polymerization of porphobilinogen [37,38]. As this metabolic process is essential for basal cell function, *HMBS* expression tends to be stably maintained across the MSC populations evaluated in this study. Moreover, *HMBS* has been recognized for its stable expression across various porcine MSCs [27]. While *GAPDH* has traditionally been the most widely used reference gene for data normalization, previous studies have demonstrated considerable instability in its expression across various feline samples, including pancreatic and spinal tissues [15,39].

To demonstrate the impact of reference gene selection on target gene quantification, we evaluated the expression of the pluripotency marker *OCT4*. As a transcriptional regulator, *OCT4* is essential for sustaining the self-renewal capacity and undifferentiated state of stem cell populations [40], and its active transcription is critical for preserving their inherent biological properties and functional stemness across various MSC sources [41]. When the expression data were normalized using *HMBS* and *TBP* identified in the present study as the most stably expressed reference genes, *OCT4* expression was significantly higher in U-MSCs than in A-MSCs (*p* < 0.05). Conversely, normalization against the unstable gene, *GAPDH*, masked this statistical significance, potentially leading to erroneous biological interpretations. These findings emphasize that relying on traditional, unvalidated housekeeping genes like *GAPDH* can compromise data reliability.

Several limitations of the present study should be acknowledged. While the current study provides consistent data regarding reference gene stability, the relatively small number of biological donors (*n* = 4) remains a limitation that may restrict broader generalizations. Although adipose and uterine tissues were collected from the same four individual cats to minimize potential inter-individual variation between tissue groups, the small number of donors may not fully represent the full extent of biological variability. Future investigations incorporating larger cohorts are warranted to confirm the stability of these internal controls. Moreover, the nine reference genes evaluated in this study were selected a priori based on previous studies, which may introduce candidate selection bias and exclude potentially more stable reference genes. Publicly available feline transcriptomic datasets could be used to pre-screen genes exhibiting consistently stable expression across MSC samples, followed by experimental qRT-PCR validation [42]. Although *OCT4* was used as a target gene to demonstrate the impact of reference gene stability on normalization, validation using additional genes would further strengthen the general applicability of the proposed reference gene selection. Nevertheless, the current results identify *HMBS* and *TBP* as the most stably expressed reference gene candidates among the nine genes in P3 undifferentiated feline A-MSCs and U-MSCs under the normal culture conditions. Further validation in larger cohorts and under diverse experimental conditions is warranted.

## 5. Conclusions

This study identifies *HMBS* and *TBP* as the most suitable reference gene candidates among nine genes for evaluating gene expression using qRT-PCR in P3 undifferentiated feline A-MSCs and U-MSCs under the normal culture conditions. Evaluation using the pluripotency marker *OCT4* demonstrated that reference gene selection could influence the interpretation of target gene expression. Normalization using *HMBS* and *TBP* revealed a significant difference in *OCT4* expression between A-MSCs and U-MSCs, whereas this difference was not detected when *GAPDH* was used. These findings identify *HMBS* and *TBP* as the two most stably expressed reference gene candidates among the nine genes evaluated under the present experimental conditions. Further validation in larger populations and under diverse experimental conditions is needed.

## Figures and Tables

**Table 1:** Information on candidate reference genes and *OCT4*.

	Information on Primers						
Gene Name (Symbol)	Sequence	Base Pair	Accession	R^2^	M	B	E
Beta-actin (*ACTB*)	F: GGACTTCGAGCAGGAGATGG	186	ON164672.1	0.986	−3.519	35.152	1.01
	R: ATGATGGAGTTGAAGGTAGTTTCG						
Glyceraldehyde-3-phosphate dehydrogenase (*GAPDH*)	F: CTGGAGAAAGCTGCCAAATACG	117	XM_006933438.4	0.984	−3.488	34.814	0.99
	R: GTTAAAGTCGCAGGAGACAACC						
Glucuronidase beta (*GUSB*)	F: GCTCCTCTATACCACACCTACC	155	NM_001009310.1	0.990	−3.481	38.915	1.00
	R: CGACTTTGCCTTCCTCATCC						
Ribosomal protein L7 (*RPL7*)	F: CTGCTGGTGATGACAAGAAAGG	152	XM_023248286.2	0.992	−3.512	35.172	1.02
	R: CTGATAGGGTGTGTTGGTTGC						
Hypoxanthine phosphoribosyl transferase 1 (*HPRT1*)	F: TCAGACTGAAGAGCTACTGTAACG	185	XM_023248905.2	0.991	−3.461	37.115	0.99
	R: TGCAACCTTGACCATCTTTGG						
TATA-box binding protein	F: AGCTTGACCTAAAGACCATTGC	80	JQ424890.1	0.999	−3.423	34.857	1.01
(*TBP*)	R: TCTCATGATAACAGCAGCAAACC						
Tyrosine 3-monooxygenase/tryptophan 5-monooxygenase activation protein zeta (*YWHAZ*)	F: CAGACTGAAGAGCTACTGTAACG	103	XM_006943327.5	0.993	−3.416	36.955	0.99
	R: TGCAACCTTGACCATCTTTGG						
Beta-2-microglobulin (*B2M*)	F: CTCCAAAGGTTCAGGTTTACTCC	184	NM_001009876.1	0.992	−3.412	38.941	1.02
	R: ACCAGAAGATAGAAAGTCCAGTCC						
Hydroxymethylbilane synthase (HMBS)	F: GTGTATTGCATGATCCTGAGACC	116	NM_001177809.1	0.997	−3.472	35.661	1.02
	R: CCCATCCTTCATAGCTGCATGC						
POU class 5 homeobox 1 (*OCT4*)	F: CACCATCGAGAATGTCAAGGC	160	NM_001173441.1				
	R: TGTTTCCCAGCAAAGATCAACC						

## Data Availability

The original contributions presented in this study are included in the article. Further inquiries can be directed to the corresponding author.

## Abbreviations

The following abbreviations are used in this manuscript:

MSC(s)	Mesenchymal Stem Cell(s)
A-MSCs	Adipose tissue-derived Mesenchymal Stem Cells
U-MSCs	Uterine tissue-derived Mesenchymal Stem Cells
qRT-PCR	Quantitative Real-Time Polymerase Chain Reaction
MIQE	Minimum Information for Publication of Quantitative Real-Time PCR Experiments
OHE	Ovariohysterectomy
*ACTB*	Beta-actin
*B2M*	Beta-2-microglobulin
*GAPDH*	Glyceraldehyde-3-phosphate dehydrogenase
*GUSB*	Beta-glucuronidase
*HMBS*	Hydroxymethylbilane synthase
*HPRT1*	Hypoxanthine phosphoribosyltransferase 1
*RPL7*	Ribosomal protein L7
*TBP*	TATA-box binding protein
*YWHAZ*	Tyrosine 3-monooxygenase/tryptophan 5-monooxygenase activation protein zeta
*OCT4*	POU Class 5 Homeobox 1
Ct	Cycle threshold
cDNA	Complementary DNA
RNA	Ribonucleic acid
FBS	Fetal bovine serum
P/S	Penicillin–Streptomycin
FITC	Fluorescein isothiocyanate
APC	Allophycocyanin
IgG	Immunoglobulin G
ANOVA	Analysis of variance
SD	Standard deviation
NF	Normalization factor
R^2^	Coefficient of determination
